# SIRT1 Activation Attenuates the Cardiac Dysfunction Induced by Endothelial Cell-Specific Deletion of CRIF1

**DOI:** 10.3390/biomedicines9010052

**Published:** 2021-01-08

**Authors:** Shuyu Piao, Ikjun Lee, Seon-Ah Jin, Seonhee Kim, Harsha Nagar, Su-jeong Choi, Byeong Hwa Jeon, Cuk-Seong Kim

**Affiliations:** 1 Department of Physiology and Medical Science, College of Medicine, Chungnam National University, Daejeon 301-747, Korea; piaoshuyu@cnu.ac.kr (S.P.); tw2622@gmail.com (I.L.); wlxlsunny@naver.com (S.K.); harsha_nagar2002@yahoo.com (H.N.); 01030028473@naver.com (S.-j.C.); bhjeon@cnu.ac.kr (B.H.J.); 2 Division of Cardiology, School of Medicine, Chungnam National University Hospital, Daejeon 301-747, Korea; drjsa@cnuh.co.kr

**Keywords:** CRIF1 protein, endothelial cells, cardiomyocytes, sirtuin 1

## Abstract

The CR6-interacting factor1 (CRIF1) mitochondrial protein is indispensable for peptide synthesis and oxidative phosphorylation. Cardiomyocyte-specific deletion of CRIF1 showed impaired mitochondrial function and cardiomyopathy. We developed an endothelial cell-specific CRIF1 deletion mouse to ascertain whether dysfunctional endothelial CRIF1 influences cardiac function and is mediated by the antioxidant protein sirtuin 1 (SIRT1). We also examined the effect of the potent SIRT1 activator SRT1720 on cardiac dysfunction. Mice with endothelial cell-specific CRIF1 deletion showed an increased heart-to-body weight ratio, increased lethality, and markedly reduced fractional shortening of the left ventricle, resulting in severe cardiac dysfunction. Moreover, endothelial cell-specific CRIF1 deletion resulted in mitochondrial dysfunction, reduced ATP levels, inflammation, and excessive oxidative stress in heart tissues, associated with decreased SIRT1 expression. Intraperitoneal injection of SRT1720 ameliorated cardiac dysfunction by activating endothelial nitric oxide synthase, reducing oxidative stress, and inhibiting inflammation. Furthermore, the decreased endothelial junction-associated protein zonula occludens-1 in CRIF1-deleted mice was significantly recovered after SRT1720 treatment. Our results suggest that endothelial CRIF1 plays an important role in maintaining cardiac function, and that SIRT1 induction could be a therapeutic strategy for endothelial dysfunction-induced cardiac dysfunction.

## 1. Introduction

Epidemiological studies have shown that cardiovascular disease is prevalent and strongly associated with the development of heart failure [1,2]. Coronary artery disease and cardiomyopathy are common reasons for heart failure, which is characterized by a decline in cardiac function. The heart is a vital pumping organ that is essential for life. It is made up of different cardiac cell types, including myocytes, endothelial cells, fibroblasts, and immune cells [3]. Cardiomyocytes and endothelial cells are considered the heart’s main cell types and are responsible for cardiac structure and function. To date, most studies have focused on cardiomyocytes as a therapeutic target for cardiac dysfunction [4,5]. Although cardiomyocytes make up the highest proportion of cardiac cells, endothelial cells are the most abundant in the heart. These not only regulate cardiomyocyte contraction but also contribute to interactions in cardiac physiology and pathologies [3,6]. Additionally, due to the unique structure of the coronary vasculature, heart endothelial cell injury leads to severe damage to other heart tissues and organs [7,8].

CR6-interacting factor1 (CRIF1) is an essential mitochondrial protein required for the synthesis and insertion of oxidative phosphorylation (OXPHOS) peptides into the inner membrane and is present in diverse mouse organs, including the brain, liver, intestines, and heart [9]. Previous studies reported that deletion of CRIF1 impaired mitochondrial oxidative function and was associated with excessive oxidative stress in different cell lines [10,11]. CRIF1-silenced adipocytes or macrophages all exhibited reduced mitochondrial oxidative function with insulin resistance and systemic inflammation [12,13]. Targeted deletion of CRIF1 in the mouse forebrain leads to neurodegeneration and mitochondrial abnormalities [14]. Thus, CRIF1 has been investigated in several studies as a potential target for mitochondrial changes and dysfunction. In their previous study, Shin et al. demonstrated that cardiomyocyte-specific deletion of CRIF1 interrupts mitochondrial structure and respiration in cardiac muscles and leads to cardiomyopathy [15]. Since the number of endothelial cells is nearly three-fold that of cardiomyocytes, and as endothelial cells control cell-to-cell communication and homeostasis, which are critical for normal cardiac function, we questioned whether impaired endothelial cells caused by mitochondrial OXPHOS dysfunction also affect cardiac function. Previously, we showed that downregulation of CRIF1 triggered endothelial dysfunction and inflammation caused by defective mitochondrial oxidative function in human umbilical vein endothelial cells [16,17]. Sirtuin 1 (SIRT1), a nicotinamide adenine dinucleotide-dependent deacetylase, ameliorates vascular endothelial dysfunction by mediating endothelial nitric oxide synthase (eNOS)/nitric oxide (NO) and scavenging oxidative stress levels, which are reportedly related to CRIF1 deletion-induced endothelial dysfunction [17]. Therefore, in this study, we evaluated whether downregulation of endothelial CRIF1 affects cardiac function and how cardiac function can be restored. To this end, we generated mice with endothelial cell-specific CRIF1 deletion that exhibited the characteristics of heart failure with impaired mitochondrial function in the heart, which was associated with decreased SIRT1 expression. We also demonstrated that SIRT1 activation by SRT1720 treatment ameliorated cardiac injury by activating eNOS and inhibiting inflammation. This represents a promising therapeutic strategy for endothelial-dysfunction-induced cardiac dysfunction.

## 2. Experimental Section

### 2.1. Mouse Studies

All experiments were approved and conducted at Chungnam National University (CNUH-019-A0016; approved date: 13 June 2019) following the guidelines of the Institutional Animal Care and Use Committee. Floxed CRIF1 (CRIF1flox/flox) mice were generated as described in a previous study [14]. Tek-Cre transgenic mice (C57BL/6J) were obtained from Jackson Laboratory (Bar Harbor, ME, USA). Floxed CRIF1 mice and Tek-Cre transgenic mice were crossed to generate Tek-CRIF1 mice. To distinguish the genotype, PCR was achieved using specific primers and extracted genomic DNA from tail snips using the kit according to the manufacturer’s instructions (TOYOBO, Iwakuni, Japan). The following primers were designed for genotyping of the mouse strains: for CRIF1-floxed allele, CRIF1-Loxp F (5′-GGGCTGGTGAAATGTGTTG-3′) and CRIF1-Loxp R (5′-TCAGCTAGGGTGGGACAGA-3′); for Tek-Cre-induced recombination, Cre F (5′-GCGGTCTGGCAGTAAAAACTATC-3′) and Cre R (5′-GTGAAACAGCATTGGTGTCACTT-3′). Mice were maintained in a controlled environment (ambient temperature 22–24 °C; humidity 50–60%; 12 h light/dark cycle).

### 2.2. Western Blot

Tissues were homogenized with RIPA lysis buffer with protease and phosphatase inhibitor cocktail. Proteins were prepared by centrifugation at 12,000 rpm for 15 min, and the supernatant was collected. Aliquots of proteins were separated by electrophoresis on SDS polyacrylamide gels and then transferred to polyvinylidene difluoride membranes (Immobilon-PSQ, Millipore, Tullagreen, Ireland). After blocking with 5% skim milk for 1 h at room temperature, the membranes were incubated overnight at 4 °C with the following specific primary antibodies. CRIF1, SIRT1, p65, phospho-p65, and VCAM-1 were all purchased from Santa Cruz Biotechnology (Santa Cruz, CA, USA). OXPHOS complex subunits (NDUFA9, SDHA, UQCRC2, and ATP5a1), and zonula occludens-1 (ZO-1) were purchased from Invitrogen (Carlsbad, CA, USA). β-actin (Sigma-Aldrich, St. Louis, MO, USA), COX-4 (Cell signaling Technology, Beverly, MA, USA), α-tubulin (R&D Systems, Minneapolis, MN, USA), collagen I (Abcam, Cambridge, MA, USA). After washing 3 times in TBST for 10 min, membranes were incubated with appropriate peroxidase-conjugated secondary antibodies for 1 h in room temperature and then washed 3 times in TBST for 10 min. Chemiluminescent signal was detected by using Super Signal West Pico or Femto Substrate from Thermo Fisher Scientific (Waltham, MA, USA).

### 2.3. Quantitative RT-PCR

Total RNA was isolated using TRIzol Reagent (Invitrogen, Carlsbad, CA, USA) based on the acid guanidinium thiocyanate–phenol–chloroform method. Total RNA concentration was determined using a SmartSpec 3000 spectrophotometer (Bio-Rad, Hercules, CA, USA). Complementary DNA was prepared from total RNA using the Maxime RT Premix kit (iNtRON Biotechnology, Gyeonggi, Korea). Quantitative real-time PCR was performed using the Prism7000 Sequence Detection System (Applied Biosystems, Foster City, CA, USA) with the Super Script III Platinum SYBR GreenOne-Step qRT-PCR Kit (Invitrogen, Carlsbad, CA, USA). The primers used for mouse VCAM-1 were sense-5′-TGAACCCAAACAGAGGCAGAG-3′ and antisense-5′-GG TATCCCATCACTTGAGCAG-3′. The primers used for mouse TNFα, IL-6, and IL-1β were as follows: TNFα sense-5′-CAGGCGGTGCCTATGTCTC-3′ and antisense-5′-CGATCACCCCGAAG TTCAGTAG-3′; IL-6 sense-5′-GCAACTGTTCCTGAACTC AACT-3′ and antisense-5′-TTGGTCC TTAGCCACTCCTTC-3′; IL-1β sense-5′-TAGTCCTTCCTACCCCAATTTCC-3′ and antisense-5′-AT CTTTTGGGGTCCGTCAACT-3′. The primers used for mouse SIRT1 were sense-5′-CTCTGAAAGT GAGACCAG TAGC-3′ and antisense-5′-TGTAGATGAGGCAAAGGTTCC-3′. The primers for mouse glyceraldehyde 3-phosphate dehydrogenase, used as the internal control, were as follows: sense-5′-ATGACATCAAGAAGGTGGTG-3′ and antisense-5′-CATACCAGGAAAATGAG CTTG-3′. Dissociation curves were monitored to check the aberrant formation of primer-dimers. The fold change in the interest gene expression was calculated by using the 2-ΔΔCt method.

### 2.4. Histological Analysis

After washing with phosphate-buffered saline, Paraffin sections of heart tissues were fixed via immersion in 4% (*w*/*v*) paraformaldehyde for 24 h and then embedded in paraffin. Paraffin sections were deparaffinized and rehydrated according to standard protocols and stained with hematoxylin-eosin. For immunofluorescence staining, heart tissues were stained with primary antibodies anti-SIRT1 (diluted in 1:100; Santa Cruz, CA, USA) and anti-ZO-1 (diluted in 1:100; Thermo Scientific, Rockford, IL, USA) overnight at 4 °C and then labeled with the secondary antibodies (diluted in 1:200; Jackson ImmunoResearch, West Grove, PA, USA) for 1 h at room temperature. Images were acquired by fluorescence microscopy. For Masson’s trichrome staining, sections were stained using a Masson’s trichrome stain kit (Polysciences, Warrington, PA, USA) following the manufacturer’s instructions.

### 2.5. Echocardiography

Mice were lightly anesthetized intraperitoneally with 15 μL/g of 2.5% avertin. Echocardiographic measurements were examined by using a Vivid E9 with an XD clear (GE) instrument with a 13-MHz linear probe (GE VIVID 7) as previously described (17). The chests of the mice were shaved and then performed by using the M-mode guide by a short-axis view of the two-dimensional mode. Changes in fractional shortening (FS) and ejection fraction (EF) were analyzed and automatically obtained as the formula.

### 2.6. SRT1720 Treatment

SRT1720 was purchased from Selleck Chemicals (Houston, TX, USA) and dissolved in DMSO at 25 mg/mL and stored at −20 °C. Before giving the injection, the SRT1720 stock solution was diluted to 20 times its original volume with PBS and delivered by intraperitoneal inject twice weekly (20 mg/kg) from 4 to 9 weeks in CRIF1 EKO mice (CRIF1 KO + SRT group). The control group mice were injected with 5% DMSO in PBS.

### 2.7. ATP Measurement

ATP content was measured in heart tissues using ATP Colorimetric Assay kit (Catalogue number: ab83355; Abcam, Cambridge, MA, USA) according to the manufacturer’s instructions.

### 2.8. Measurement of Mitochondrial Membrane Potential and Mitochondrial ROS

Mitochondria used for mitochondrial membrane potential measurement were isolated from heart tissues using Mitochondria Isolation Kit (Catalogue number: 89801; Thermo Fisher Scientific Inc., Waltham, MA, USA). The isolated mitochondria were incubated with tetramethyl rhodamine ethyl ester (TMRE) (Invitrogen, Carlsbad, CA, USA). dye at 37 °C for 15 min in the dark. Following the incubation period, each sample was washed twice with PBS and examined by using the Fluoroskan Ascent fluorescence reader at excitation 530 nm and emission 590 nm, respectively. For the mitochondrial ROS measurement, the isolated heart mitochondria were incubated with 3 μM MitoSOX red fluorescence (Invitrogen, Carlsbad, CA, USA) at 37 °C for 15 min in the dark. To lessen fluorescence background, each sample was washed twice with PBS and then detected by using the Fluoroskan Ascent fluorescence reader at excitation 530 nm and emission 590 nm, respectively.

### 2.9. SIRT1 Activity

The nuclear and cytoplasmic fraction of heart tissue was separated using the NE-PER Nuclear and Cytoplasmic Extraction Reagents Kit (Catalogue number:18833; Thermo, Fisher Scientific, Rockford, IL, USA). SIRT1 activity was determined in the nuclear extract from heart tissues by using SIRT1 fluorometric assay kit (Catalogue number: ab156065; Abcam) according to the manufacturer’s instructions. The fluorescence intensity (Ex355nm/Em460nm) was measured at 2 min intervals for 60 min using the Fluoroskan Ascent fluorescence reader.

### 2.10. Isolation of Mouse Coronary Endothelial Cells

To purify cardiac endothelial cells, 9-week-old mice were used for our experiments. Mice hearts were removed from the chest cavity of mice and then minced into small pieces. After digesting the heart using collagenase I (LS004196, Worthington, Lakewood, NJ, USA), cells were washed with PBS. Following, a magnetic-activated cell sorting (MACS) system was used to isolate cardiac endothelial cells according to the manufacturer’s instructions. Both of CD31 MicroBeads (cat no. 130-097-418) and CD45 MicroBeads (cat no. 130-052-301) used for MACS were obtained from Miltenyi Biotec Inc. (Bergisch Gladbach, Germany). Cardiac endothelial cells were identified as CD45-negative cells and CD31-positive cells, which were used for analysis.

### 2.11. Nitrite and Nitrate Measurements

Nitrite and nitrate, which are the final products of nitric oxide (NO), were measured to quantify the formation of NO. Nitrate/nitrite colorimetric assay kit was purchased from Abcam (Cambridge, MA, USA). After deproteinizing mouse serum using a 10-kDa cutoff filter, a colorimetric assay was performed following the manufacturer’s instructions. The levels of NO were normalized by subtracting the background colorimetric to obtain the total nitrite/nitrate amount as described previously [18].

### 2.12. ROS Measurement

Fluorescent HPLC method was used for analyzing ROS content by detecting the dihydroethidium (DHE) oxidation product 2-hydroxyethidium (2-EOH) [19,20,21]. Heart tissues were incubated in Krebs bicarbonate buffer (110 mM NaCl, 4.7 mM KCl, 1.9 mM CaCl_2_, 1.2 mM MgSO_4_, 1.03 mM K_2_HPO_4_, 25 mM NaHCO_3_, 11.1 mM glucose) contained DHE (10 μM) at 37 °C. After 1 h incubation, tissue was washed with Krebs buffer and homogenized with 1:1 solution of acetonitrile and distilled water. The homogenized sample was sonicated and centrifuged. Supernatant was collected for fluorescent HPLC-based 2-EOH assay (1290 series, Agilent). Samples or 2-EOH standards were injected to C-18 column (ZORBAX, Agilent, 2.1 × 150 mm) through mobile phase (37% acetonitrile and 0.1% trifluoroacetic acid, 0.5 mL/min). 2-EOH florescent signal was detected at 580 nm (emission) and 480 nm (excitation).

### 2.13. Statistical Analysis

Statistical analysis was performed by using GraphPad Prism 6 (GraphPad Software Inc., San Diego, CA, USA). Data are expressed as means ± standard error of mean (SEM). Differences between two groups were evaluated using *t*-tests. For multiple comparisons, one-way analysis of variance (ANOVA) was performed following by an appropriate multiple comparison test. *p*-Values less than 0.05 were considered statistically significant. Data are representative of at least three independent experiments.

## 3. Results

### 3.1. Generation of Endothelial Cell-Specific Deletion of CRIF1 in Mice

**Figure 1:**
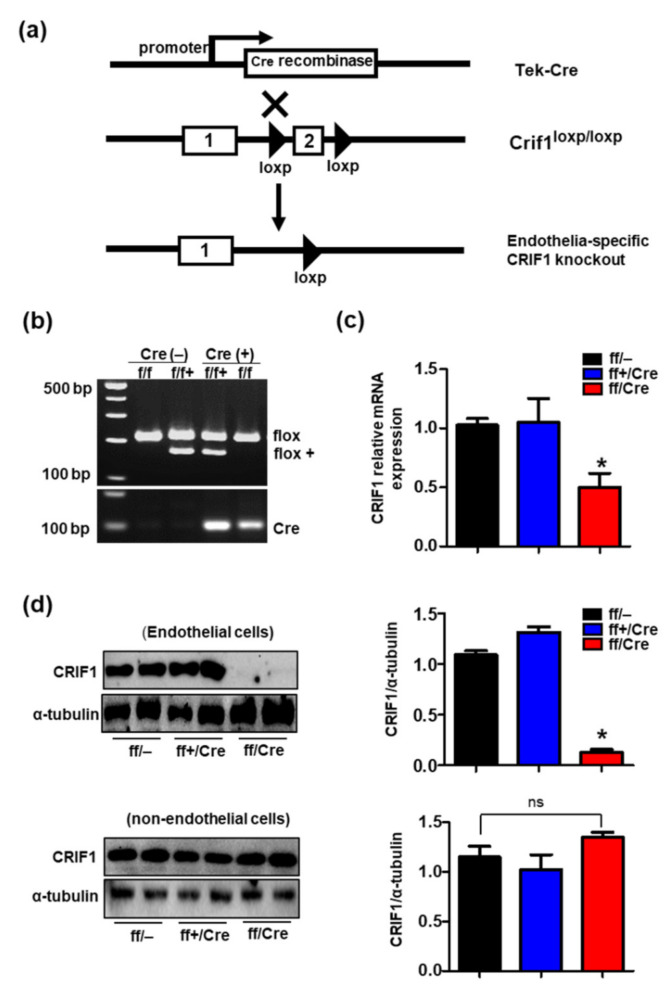
Generation of endothelial cell-specific deletion of CR6-interacting factor1 (CRIF1) in mice. (**a**) Schematic diagram showing endothelial deletion of CRIF1 gene target strategy. The numbers indicate exons of CRIF1, and the triangles show the loxp sites. (**b**) Representative images of PCR genotyping for CRIF1^loxp^ and Cre primers are shown. WT: flox/flox/Cre– and flox/flox+/Cre–; Hetero: flox/flox+/Cre; CRIF1: flox/flox/Cre. (**c**) mRNA expression was examined by qPCR in the cardiac endothelial cells of mice. (**d**) Western blotting analysis of CRIF1 in the endothelial and non-endothelial cells isolated from the hearts of mice. CRIF1 protein levels were quantified by densitometric analysis. The data are presented as means ± SEM of at least three independent experiments (*n* = 5 mice in each group; ns: not significant; * *p* < 0.05 vs. ff/– group).

Previous studies reported that deletion of CRIF1 in various cell types reduced oxidative capacity and triggered mitochondrial dysfunction [12,13]. To investigate whether the defective endothelial cells caused by mitochondrial OXPHOS also affected heart function, we generated mice with endothelial cell-specific deletion (EKO) of CRIF1 by crossing CRIF1loxp/loxp C57BL6/J mice with Tek-Cre transgenic mice (Figure 1a). The genotype was confirmed by polymerase chain reaction of the loxp gene and Cre recombinase (Figure 1b). Both mRNA and protein expression of CRIF1 was decreased in endothelial cells isolated from the heart of ff/Cre (CRIF1 EKO) mice compared with the control mice ff/– (WT) (Figure 1c,d). In addition, the protein expression of CRIF1 in non-endothelial cells showed no differences between the ff/– (WT) and ff/Cre (CRIF1 EKO) mice (Figure 1d). However, there was no difference in CRIF1 expression between the control ff/– (WT) and ff+/Cre (hetero) mice. These results confirmed that CRIF1 was specifically deleted in the endothelial cells of CRIF1 EKO mice.

### 3.2. Endothelial Cell-Specific Deletion of CRIF1 Causes Cardiac Defects

**Figure 2:**
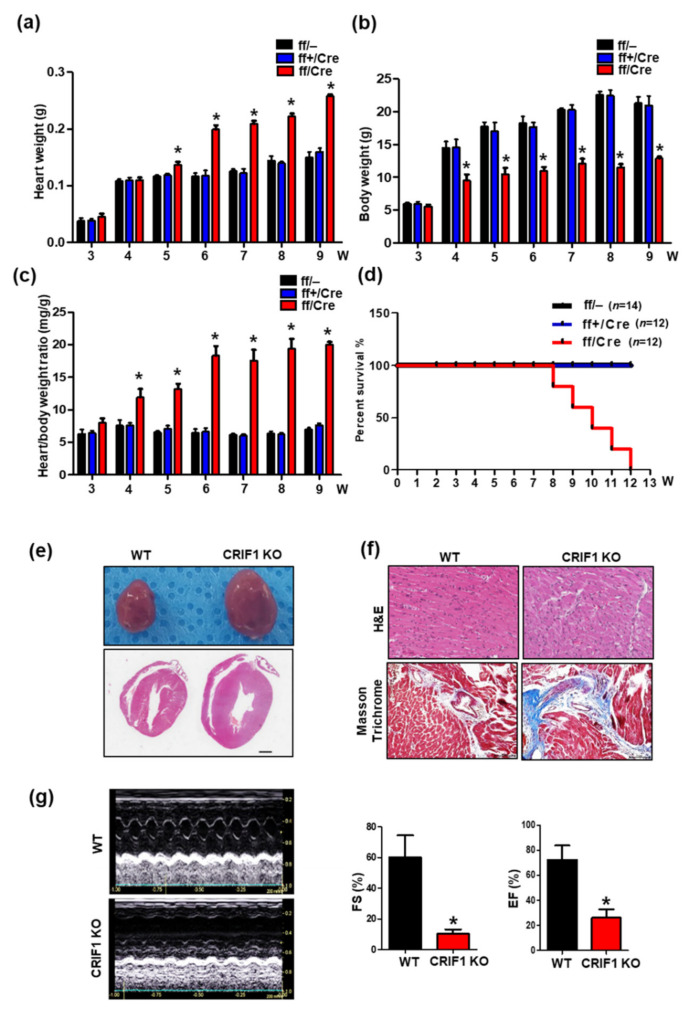
Endothelial cell-specific deletion of CRIF1 causes cardiac defects. (**a**) Body weight and (**b**) heart weight and (**c**) heart/body weight of 3 to 9-week-old mice (*n* = 4). (**d**) Survival rate of WT (ff/–), hetero (ff+/Cre), and CRIF1 EKO mice (ff/Cre) (*n* = 12~14 mice in each group). (**e**) Representative whole mount hearts (top) and hematoxylin and eosin staining of four chamber histological sections (bottom) and from the hearts of mice. (Scale bar, 1 mm) (**f**) Representative H&E staining and Masson’s trichrome staining of high-magnification sections of the hearts of mice. (**g**) Representative M-mode echocardiograms from WT (up) and CRIF1 EKO (down). Percentage of FS and EF determined from echocardiography (*n* = 4 mice in each group). All representative images were examined from 9-week-old mice. All data are presented as means ± SEM of at least three independent experiments. * *p* < 0.05 vs. WT group.

We examined cardiac function and morphology to determine the effect of endothelial CRIF1 deletion on the heart. Bodyweight, heart weight, and heart-to-bodyweight ratio were detected in ff/– (WT), ff+/Cre (hetero), and ff/Cre mice (KO), respectively. The heart weight and heart-to-bodyweight ratio were increased from 5-week-old and 4-week-old CRIF1 EKO mice compared with age-matched WT and hetero mice, respectively (Figure 2a,c). The bodyweight was significantly lower in 4-week-old KO mice compared with the other two groups (Figure 2b). All CRIF1 EKO mice died before reaching the age of 12 weeks; in contrast, none of the WT and hetero mice died during this period (Figure 2d). The heart was significantly larger in CRIF1 EKO mice compared with the control mice at nine weeks old (Figure 2e). Hematoxylin and eosin staining of heart sections showed an impaired structure in CRIF1 EKO mice (Figure 2f; upper). Masson’s trichrome staining also detected high levels of collagen and elastin fibers (blue color) in the CRIF1 EKO mice (Figure 2f; lower). Next, to evaluate the cardiac performance of CRIF1 EKO mice, we performed transthoracic echocardiography in 9-week-old mice. Fractional shortening (FS) and ejection fraction (EF) measurements were used to evaluate the left ventricular function. As shown in Figure 2g, the mean FS percentage was significantly lower in CRIF1 EKO mice than in WT mice (10.19 ± 2.90% vs. 60.06 ± 14.38; *p* < 0.05). Consistent with these results, the mean EF percentage was also significantly lower in CRIF1 EKO mice than in WT mice (26.09 ± 6.77% vs. 72.17 ± 11.50%; *p* < 0.05). These results suggest that endothelial cell-specific deletion of the CRIF1 gene induces severe cardiac dysfunction and leads to premature death.

### 3.3. Endothelial Cell-Specific Deletion of CRIF1 Causes Mitochondrial Dysfunction in the Heart

**Figure 3:**
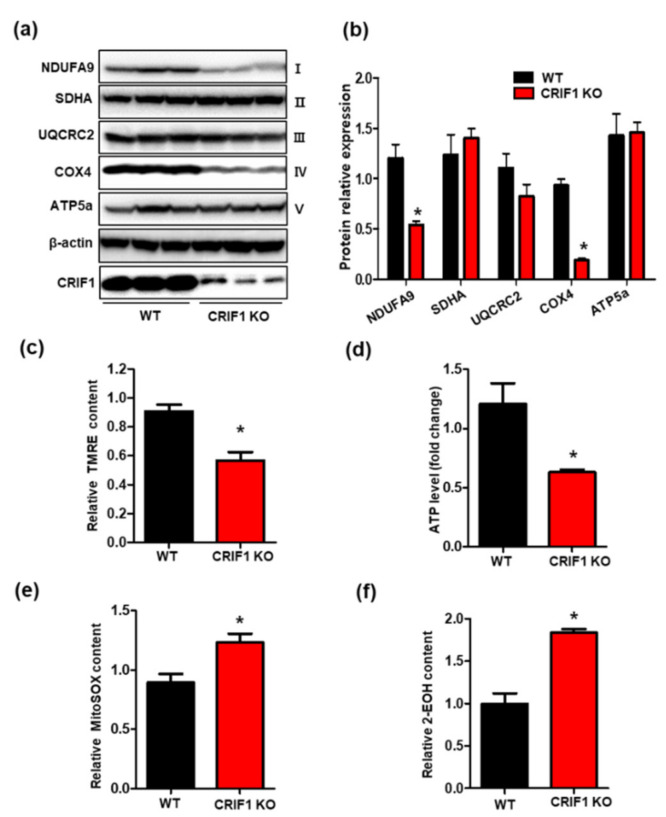
Endothelial cell-specific deletion of CRIF1 causes mitochondrial dysfunction in the heart. (**a**) Western blot analysis for mitochondrial oxidative phosphorylation (OXPHOS) complex subunits in the heart tissues of mice. (**b**) OXPHOS subunits protein levels were quantified by densitometric analysis. (**c**) Tetramethyl rhodamine ethyl ester (TMRE) and (**d**) cardiac ATP levels were determined by using a colorimetric/fluorometric assay kit. (**e**) Mitosox-red fluorescence intensity. (**f**) Relative 2-hydroxyethidium (2-EOH) fluorescence intensity were measured by fluorescence reader. All data are presented as means ± SEM of at least three independent experiments (*n* = 5 mice in each group; * *p* < 0.05 vs. WT group).

As CRIF1 is indispensable for the biogenesis of OXPHOS subunits, we evaluated protein expression of the following OXPHOS complex subunits: complex I (NDUFA), complex II (SDHA), complex III (UQCRC2), complex IV (COX4), and complex V (ATP5a) in cardiac tissues. As shown in Figure 3a,b, protein expression of complexes I and IV was significantly reduced in the CRIF1 EKO group compared with the WT group. The mitochondrial membrane potential is generated by OXPHOS complex subunits (complexes I, III, and IV), which serve as intermediate energy storage units for ATP synthesis. Since we observed decreased protein expression of complexes I and IV in the CRIF1 EKO group, we investigated mitochondrial membrane potential and ATP production, respectively. Tetramethyl rhodamine ethyl ester (TMRE) is a fluorescent dye used to measure mitochondrial membrane potential. Our results showed decreased fluorescence from TMRE and reduced ATP production in the CRIF1 EKO group (Figure 3c,d), suggesting impaired OXPHOS complex generation and mitochondrial function in endothelial cells with CRIF1 deletion. Considering that mitochondria are a source of excessive reactive oxygen species (ROS) production, we examined mitochondrial and cytosolic ROS levels in the CRIF1 EKO mice group. We found increased levels of either mitochondrial or cytosolic ROS in cardiac tissues of the CRIF1 EKO group compared with the WT group (Figure 3e,f). Taken together, these results suggest that loss of CRIF1 expression in endothelial cells contributes to dysfunctional mitochondria and elevated oxidative stress.

### 3.4. Increased SIRT1 Expression and Activation in CRIF1 EKO Mouse Hearts

**Figure 4:**
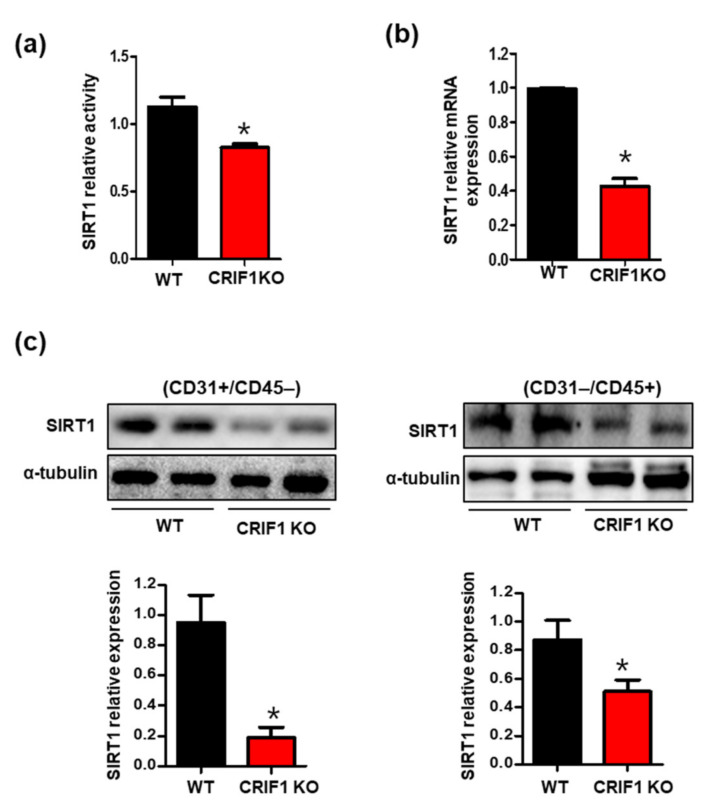
Decreased sirtuin 1 (SIRT1) expression and activation in CRIF1 EKO mouse hearts. (**a**) SIRT1 deacetylase activity in heart homogenates. (**b**) SIRT1 mRNA levels in the heart tissues. (**c**) Cardiac endothelial cells were separated from the heart tissues using anti-CD31 and anti-CD45 magnetic beads. Endothelial (CD31+/CD45−) and non-endothelial (CD31−/CD45+) SIRT1 protein expression were determined by Western blotting. α-tubulin was used as the internal control. The data are presented as means ± SEM of at least three independent experiments. (*n* = 5 mice in each group; * *p* < 0.05 vs. WT mice).

Previously, we showed that CRIF1 downregulation decreased SIRT1 expression via mitochondrial ROS in vascular endothelial cells [17]. Furthermore, other studies revealed that SIRT1 plays a crucial role in maintaining cardiac mitochondrial integrity [22]. To examine whether SIRT1 expression was affected by mitochondrial dysfunction and increased ROS levels in cardiac tissues with endothelial cell-specific deletion of CRIF1, we first measured SIRT1 activity. CRIF1 EKO mouse hearts showed a 30% reduction in SIRT1 activity compared with the WT mice (Figure 4a). There were also decreased mRNA expression levels of SIRT1 in CRIF1 EKO mice (Figure 4b). To detect SIRT1 protein expression in different cell types accurately, we purified CD31+/CD45− endothelial cells, as well as non-endothelial CD31−/CD45+ cells, including cardiomyocytes, fibroblasts, and vascular smooth muscle cells, using the magnetic-activated cell sorting system. Both the endothelial and non-endothelial cardiac cells exhibited a remarkable reduction in the SIRT1 level in the CRIF1 EKO group (Figure 4c), indicating that endothelial CRIF1 deletion inhibited SIRT1 protein levels, not only in endothelial cells but also in neighboring cells.

### 3.5. SIRT1 Activation Alleviates the Cardiac Dysfunction Induced by Endothelial Cell-Specific CRIF1 Deletion

**Figure 5:**
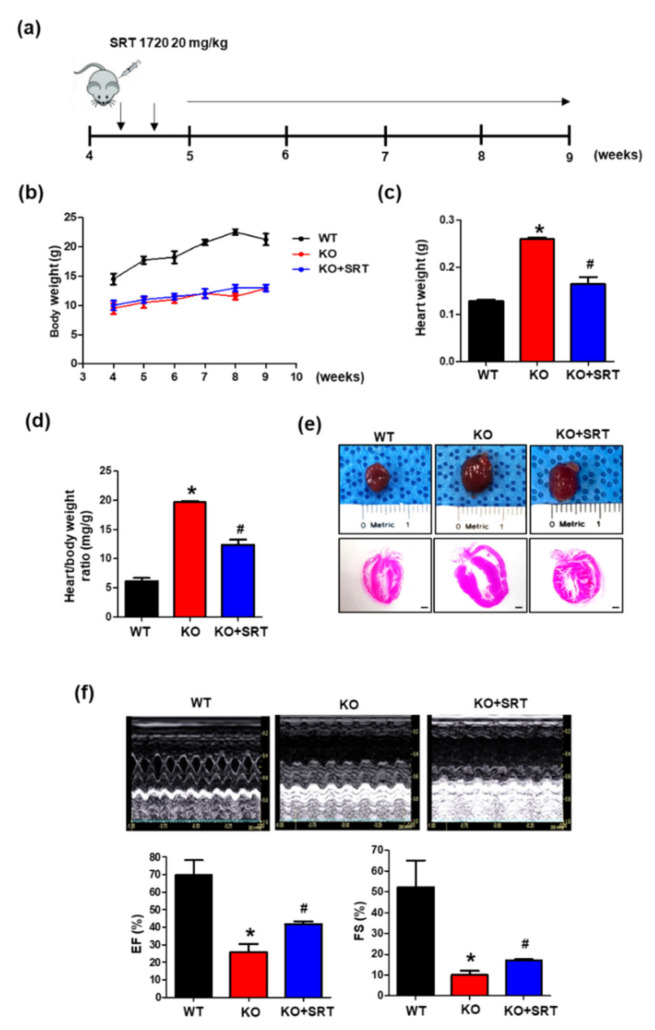
SIRT1 activation alleviates the cardiac dysfunction induced by endothelial cell-specific CRIF1 deletion. (**a**) Experimental treatment scheme. Mice were administered with SRT1720 (20 mg/kg) via intraperitoneal injection. twice per week from 4 to 9 week. (**b**) Line graph showing changes in body weight at weekly time points (4 to 9 weeks). (**c**) Heart weight and (**d**) heart/body weight of 9-week-old mice in WT, CRIF1 EKO, and CRIF1 EKO with SRT1720 treatment groups. (**e**) Representative whole mount hearts (top), H&E staining of the hearts of mice (Scale bar, 1 mm). (**f**) Representative M-mode echocardiograms and percentage of EF and FS determined from echocardiography. All data are presented as means ± SEM of at least three independent experiments (*n* = 4~7 mice in each group; * *p* < 0.05 vs. WT mice. # *p*< 0.05 vs. CRIF1 EKO mice).

SIRT1 can protect the heart from progressive cardiac failure [23], and SIRT1 showed reduced expression in CRIF1 EKO cardiac tissues. To confirm whether SIRT1 treatment also restored cardiac function in mice with endothelial cell-specific deletion of CRIF1, the SIRT1 activator SRT1720 (20 mg/kg) was injected intraperitoneally twice weekly from the age of 4 to 9 weeks (Figure 5a). We verified that SRT1720 treatment restored SIRT1 protein levels and SIRT1 activity but had no effect on SIRT1 mRNA expression (Supplementary Figure S1a). Further evaluation of cardiac function was assessed in 9-week-old mice. There was no significant difference in bodyweight between the SRT1720-treated group and WT group at any time point examined (Figure 5b). However, SRT1720 treatment reduced the heart weight and heart-to-bodyweight ratio by 36 and 37% compared with the CRIF1 EKO group (Figure 5c,d). Consistent with these results, we observed reduced heart sizes in the SRT1720-treated group compared with the CRIF1 EKO group (Figure 5e). Echocardiography results showed that SRT1720 treatment significantly increased the FS percentage compared with the CRIF1 EKO mice (16.97 ± 0.55% vs. 10.01 ± 2.06%; *p* < 0.05) (Figure 5f). Consistent with these results, the mean EF percentage was also higher in the SRT1720-treated mice than in the WT mice (41.79 ± 1.58% vs. 25.66 ± 4.81%; *p* < 0.05) (Figure 5f). These results suggest that SIRT1 activation may improve cardiac function in mice with endothelial cell-specific deletion of CRIF1.

### 3.6. SIRT1 Activation Upregulates the Expression of Zonula Occludens-1 (ZO-1) and Collagen I

**Figure 6:**
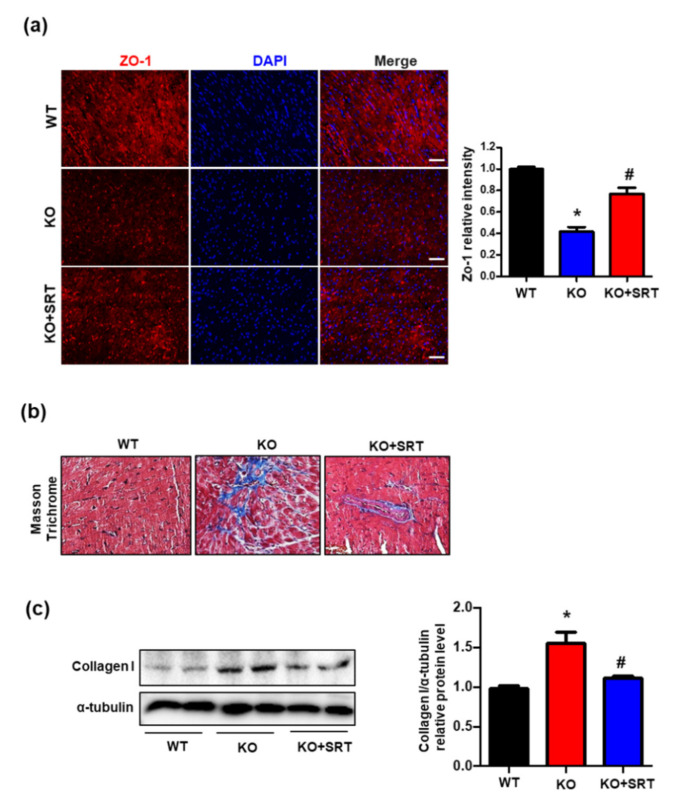
SIRT1 activation upregulates the expression of ZO-1 and collagen I. (**a**) Representative immunofluorescent staining images of ZO-1 (red) and nuclei (blue) of mouse hearts. Quantified ZO-1 expression was normalized to the WT group (Scale bars, 200 µm). (**b**) Representative Masson’s trichrome staining of high-magnification sections of the hearts of mice. (**c**) Cardiac collagen I protein expression were determined by Western blotting. α-tubulin was used as the internal control. Densitometric analysis of collagen I protein level is shown. The data are presented as means ± SEM of at least three independent experiments. (*n* = 5 mice in each group; * *p* < 0.05 vs. WT mice. # *p* < 0.05 vs. CRIF1 EKO mice).

ZO-1 is a critical tight junction protein that is expressed in endothelial cells and cardiac myocytes and is essential for endothelial barrier formation by regulating microvascular permeability and, therefore, cardiomyocyte function [24]. Immunofluorescent analyses suggested an effect of intraperitoneal SRT1720 injection on ZO-1 expression. ZO-1 fluorescence intensity was decreased in the CRIF1 EKO group compared with the WT group; however, treatment with SRT1720 partially restored the expression of ZO-1, thus enhancing the endothelial barrier function (Figure 6a). Additionally, dysfunction of endothelial cells contributes to cardiac fibrosis via accumulation of extracellular matrix proteins [25,26]. Masson’s trichrome staining showed lower levels of collagen and elastin fibers in the SRT1720-treated mice than in WT mice (Figure 6b). We examined changes in the protein expression of collagen I as an indicator of collagen deposition. Collagen I expression in CRIF1 EKO hearts was significantly reduced in the SRT1720-treated group (Figure 6c). In summary, SIRT1 activation rescued the disrupted endothelial barrier and decreased cardiac fibrosis formation in mice with endothelial cell-specific CRIF1 deletion.

### 3.7. SIRT1 Activation Mediates eNOS/NO Activation and ROS Levels

**Figure 7:**
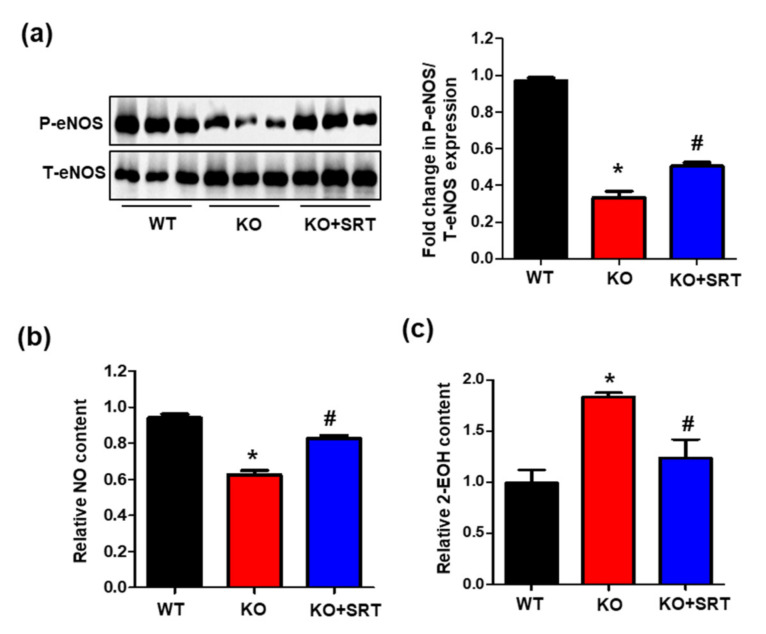
SIRT1 activation mediates eNOS/NO activation and ROS levels. (**a**) Western blot analysis for phosphorylation at serine 1117 of eNOS, total eNOS, and α-tubulin in the heart tissues of mice. The protein expression levels were quantified by densitometric analysis (right). (**b**) Serum levels of nitrite and nitrate levels were examined in mice. (**c**) Relative 2-EOH fluorescence intensity measured the heart tissues of mice. The data are presented as means ± SEM of at least three independent experiments (*n* = 5 mice in each group; * *p* < 0.05 vs. WT mice. # *p* < 0.05 vs. CRIF1 EKO mice).

NO derived from cardiac endothelial cells and cardiomyocytes (4:1) [27] modulates ventricular pump function and contractility as well as exerts cardioprotective effects [28,29,30]. Reduced SIRT1 expression leads to increased oxidative stress and reduced eNOS/NO production [31,32]. Consistent with these studies, downregulated SIRT1 expression in CRIF1 EKO mice led to reduced eNOS phosphorylation and NO production and excessive ROS levels. Next, we investigated whether SRT1720 treatment affected eNOS/NO and ROS expression. The results showed that the eNOS phosphorylation level was reduced to ~33% in CRIF1 EKO mouse hearts compared with the WT mice, and then recovered to ~50% after SRT1720 injection (Figure 7a). In parallel with the changes in eNOS phosphorylation, SIRT1 activation also increased NO production compared with the CRIF1 EKO group (Figure 7b). In addition, the increase in ROS levels was significantly prevented by SRT1720 treatment (Figure 7c). Therefore, these results suggest that activation of SIRT1 by SRT1720 partially promotes eNOS/NO production and inhibits ROS generation in CRIF1 EKO mice.

### 3.8. SIRT1 Activation Inhibits the Levels of Inflammatory Mediators in Heart Tissues

**Figure 8:**
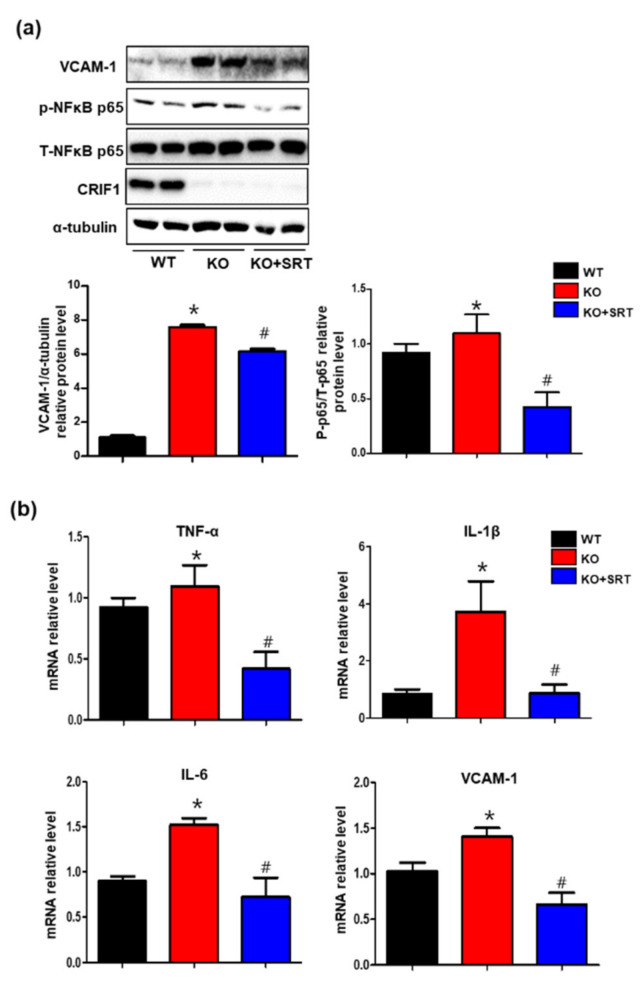
SIRT1 activation inhibits the levels of inflammatory mediators in heart tissues. (**a**) VCAM-1, p-p65, and total p65 protein expression in the heart tissues were detected by Western blotting. VCAM-1 and p-p65 protein levels were quantified by densitometric analysis. (**b**) TNFα, IL-1β, IL-6, and VCAM-1 mRNA levels in the heart tissues were quantified using qPCR. All data are presented as means ± SEM of three independent experiments (*n* = 5 mice in each group; * *p* < 0.05 vs. WT mice. # *p* < 0.05 vs. CRIF1 EKO mice).

Previously, we reported that SIRT1 mediates CRIF1 deletion-induced endothelial inflammation by activating the transcription factor NFκB and inflammatory mediators (TNF-α, IL-1β, IL-6, and VCAM-1) in human umbilical vein endothelial cells [16]. To elucidate the effect of SRT1720 on inflammation in cardiac tissues, we evaluated NFκB activation and the production of inflammatory mediators (TNF-α, IL-1β, IL-6, and VCAM-1) in each group. Western blotting showed that the increase in the VCAM-1 and phosphorylated p65 level in CRIF1 EKO mice was reduced by SRT1720 treatment (Figure 8a), suggesting that SIRT1 mediates NFκB activation in CRIF1 EKO mice. Furthermore, SRT1720-treated CRIF1 EKO mice exhibited lower TNF-α, IL-1β, IL-6, and VCAM-1 mRNA levels compared with CRIF1 EKO mice (Figure 8b). These findings suggest that SIRT1 activation reduced cardiac inflammatory processes by inhibiting inflammatory mediators, as seen in the CRIF1 EKO mice.

## 4. Discussion

Our results revealed a vital role of CRIF1 in endothelial cells in maintaining cardiac function. Deletion of CRIF1 in endothelial cells using the Tek-Cre transgene led to premature death, increased heart size, and reduced cardiac function. In addition, deletion of endothelial CRIF1 resulted in progressive mitochondrial OXPHOS dysfunction accompanied by reduced SIRT1 expression in the heart. However, SIRT1 activation by SRT1720 partially restored cardiac function and inhibited ROS and inflammation levels.

Earlier evidence focused on the impact of cardiomyocytes on cardiac function [33,34], but recent studies have focused on endothelial cells, because they not only regulate vascular tone by controlling the contractile responses of cardiomyocytes, but also contribute to cardiac remodeling and development [25,35,36]. Endothelial cell damage can occur in various organs of the vascular bed; however, damaged endothelial cells within the heart lead to more significant injury to the heart muscle than to other tissues due to the unique structure of the coronary vasculature. In addition, the heart is a vital organ with abundant capillaries that provide oxygen and nutrients to the heart tissues, and capillary endothelial cells and cardiomyocytes are located nearby (within 1 µm), which explains why endothelial dysfunction can have a significant influence on the health and function of cardiomyocytes [37]. Furthermore, endothelial cells also affect multiple cardiac functions and intercellular communication via small molecules and peptides. Deletion of Jag1 in endothelial cells disrupted endothelial-to-mesenchymal transition and induced heart defects in both embryonic and adult mice [38]. Ino80 endothelial-deficient hearts develop congenital heart diseases due to disrupted coronary angiogenesis [39]. Moreover, defects in endothelial S1pr1 worsened cardiac hypertrophy and fibrosis in the myocardium via regulation of the AKT/eNOS pathway [40]. In this study, we targeted CRIF1 to induce endothelial mitochondrial dysfunction and focused on its effects on maintaining cardiac function. Our results are similar to previous experimental studies in which endothelial cell-specific deletion of CRIF1 was implicated in the pathogenesis of cardiovascular diseases, leading to an enlarged heart and reduced cardiac function. Our findings show that CRIF1 plays an essential role in cardiac function, homeostasis, and development. However, our study differed from that by Shin et al. [15] in that CRIF1 deletion in endothelial cells provoked more severe heart failure symptoms and a high mortality rate. The heart-to-bodyweight ratio in endothelial cell-specific CRIF1 deleted mice was nearly 10% higher compared with cardiomyocyte-specific CRIF1-deleted mice. The EF and FS values were nearly three-fold lower in mice with endothelial cell-specific CRIF1 deletion than in mice with cardiomyocyte-specific CRIF1 deletion. In addition, endothelial cell-specific CRIF1 deleted mice have a much shorter life span compared with cardiomyocyte-specific CRIF1-deleted mice. This suggests that CRIF1 plays a more essential role in regulating cardiac function in endothelial cells than in cardiomyocytes. Rhee et al. reported that deletion of Ino80 in endothelial cells prevented ventricular compaction and coronary angiogenesis during mouse heart development, ultimately causing heart disease [39]. Our results showed no significant differences in heart weight or bodyweight between the control and CRIF1 endothelial cell-specific deletion hearts until 3 weeks after birth, indicating that CRIF1 deletion did not affect the growth or development of heart tissues. However, whether loss of CRIF1 expression is involved in triggering organ maturation during embryonic cardiac development needs to be investigated in future studies.

Examination of mitochondrial OXPHOS function identified CRIF1 as a potential target for investigation. We observed lost expression of OXPHOS complexes I and IV along with damaged membrane potential, decreased ATP production, increased ROS production, and impaired mitochondrial bioenergetics associated with cardiac dysfunction, possibly due to the high energy demands of the heart. As a crucial protein in cellular oxidative stress and metabolic regulation, SIRT1 has been reported to improve mitochondrial function and ameliorate oxidative stress [41]. Moreover, mice with cardiac-specific SIRT1 deletion displayed markedly reduced cardiac function accompanied by hypertrophy and fibrosis [42]. Abnormal cardiac function in diabetic cardiomyopathy mice was also associated with reduced SIRT1 expression [43]. We investigated whether the expression of SIRT1 was also modulated in CRIF1 EKO mouse heart tissues. Since endothelial cells affect cardiac remodeling and functioning via communication with cardiomyocytes and other surrounding cells, we separated endothelial cells from non-endothelial cells using CD31/CD45 beads before examining the expression of SIRT1. Interestingly, the expression of SIRT1 in endothelial and non-endothelial cells was markedly reduced in the CRIF1 EKO mice, suggesting that endothelial cell-specific deletion of CRIF1 not only modulates the levels of SIRT1 in endothelial cells but in other cardiac cells. In addition, the expression of the tight junction protein ZO-1 was reduced, which could induce microvascular permeability and impair cellular communication, including communication among endothelial cells, cardiomyocytes, and other cardiac cells. This further proves that crosstalk among different cardiac cells affects the complex environment of cardiac tissues.

Based on the increasingly important role of SIRT1 in cardiac disease, SIRT1 activators (resveratrol and SRT1720) have been shown to exert multifunctional protective roles against diabetic cardiomyopathy and other cardiac diseases via antioxidant and anti-inflammatory effects [43,44]. Additionally, intraperitoneal injection of SRT1720 attenuated experimental osteoarthritis by reducing the levels of apoptotic and inflammatory markers [45]. Abundant evidence has shown that endothelial cell dysfunction is related to abnormal coronary microcirculation, impaired cell signaling, and reduced angiogenesis. These effects eventually induce elevated oxidative stress, inflammation, cardiac fibrosis, and even heart failure [46]. Following previous research, SIRT1 activation may ameliorate cardiac function by regulating changes during oxidative stress, inflammation, and other adverse factors. To verify this hypothesis, we activated SIRT1 by injecting SRT1720 for 6 weeks, which resulted in significantly enhanced cardiac function and reduced heart weight and size in CRIF1 EKO mice but had no effect on bodyweight. This study demonstrated that activation of SIRT1 by SRT1720 exerted beneficial effects to improve the cardiac dysfunction induced by endothelial cell-specific deletion of CRIF1.

Endothelial cells, which form structural barriers, not only modulate the contraction of cardiac muscle cells via the release of NO, but also exert anti-inflammatory roles and protect against oxidative damage. Therefore, in this study, CRIF1 deletion in endothelial cells disturbed the cardiac tissue microenvironment and impaired NO production, resulting in generation of excessive ROS and inflammatory mediators. Although most NO in the heart is produced by endothelial eNOS within endothelial cells, some NO is generated by cardiomyocytes [27]. Disrupted NO production and elevated levels of ROS are considered indicators of cardiovascular disease. Previous studies also support the idea that SIRT1 activation contributes to NO activation and antioxidant and anti-inflammatory activities [47,48]. Therefore, in this study, we showed that SRT1720 affects the levels of NO production, inflammatory mediators, and oxidative stress. Consistent with these findings, the SIRT1 agonist SRT1720 also attenuated the levels of NO production, inflammatory mediators, and ROS in the cardiac tissues of mice with endothelial cell-specific deletion of CRIF1, which means that SIRT1 also had protective effects in endothelial OXPHOS dysfunction-induced cardiac defects. This study has some limitations. First, isolation of all the different cell types in cardiac tissues to show communication among cardiac cells is challenging. Future studies are needed to clarify the mechanism. Second, although intraperitoneal injection of SRT1720 showed improved effects on heart function, SRT1720 may have exerted local or systemic effects on other cells.

## 5. Conclusions

Our results showed that reduced endothelial OXPHOS function after deletion of CRIF1 led to severe impairment in cardiac function associated with reduced NO and increased inflammatory mediator levels, which partially improved after SRT1720 treatment. Therefore, our findings suggest that endothelial CRIF1 plays an essential role in maintaining cardiac function, and that SIRT1 is a promising therapeutic strategy for endothelial OXPHOS dysfunction-induced cardiac diseases.

## Data Availability

The data presented in this study are available on request from the corresponding author.

## Supplementary Materials

The following are available online at https://www.mdpi.com/2227-9059/9/1/52/s1, Figure S1: SIRT1 activator SRT1720 upregulates SIRT1 expression in the heart tissues.
