# Supplementary material for: SIRT1 Activation Attenuates the Cardiac Dysfunction Induced by Endothelial Cell-Specific Deletion of CRIF1
Source: Biomedicines. 2021 Jan 8;9(1):52. doi: 10.3390/biomedicines9010052 (PMC7827654; doi:10.3390/biomedicines9010052)
Supplement: Supplementary file 1 [file biomedicines-09-00052-s001.pdf]

Supplementary Figure 1.

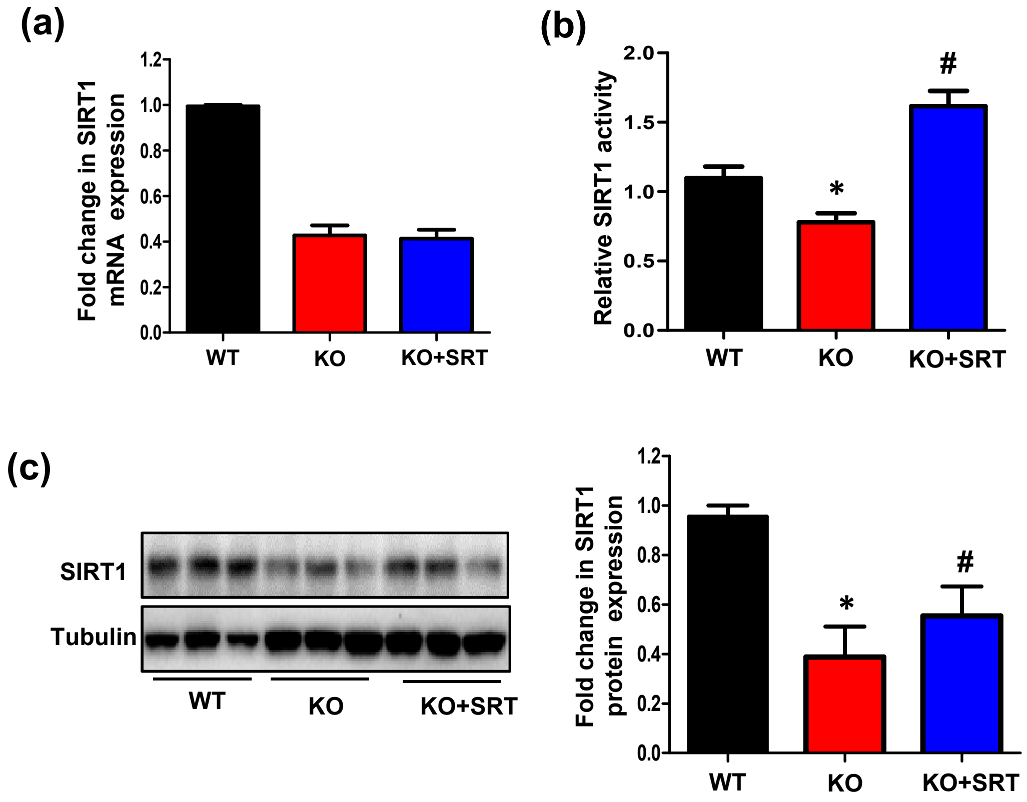

Figure 1 (a) SIRT1 mRNA levels in the heart tissues were quantified using qPCR. (b) SIRT1 deacetylase activity in heart homogenates. (c) SIRT1 protein expression in the heart tissues were determined by western blotting. α-tubulin was used as the internal control. Densitometric analysis of SIRT1 protein levels is shown. All data are presented as means ± SEM of three independent experiments. *p < 0.05 vs. WT mice. #p < 0.05 vs. CRIF1 EKO mice.
